# Adipose-tumor crosstalk in colorectal cancer: Identifying (Epi)genetic biomarkers for tumor progression and cachexia

**DOI:** 10.1038/s41419-025-07982-6

**Published:** 2025-10-06

**Authors:** Ada Pesapane, Lucia Capasso, Maria Rosaria Del Sorbo, Lucia Scisciola, Teresa Troiani, Donato Mele, Martina Franzese, Armando Puocci, Giovanni Tortorella, Surina Surina, Giacomo Fuschillo, Francesco Caraglia, Vincenzo De Falco, Lucio Selvaggi, Rosaria Anna Fontanella, Fortunato Ciardiello, Francesco Selvaggi, Lucia Altucci, Giuseppe Paolisso, Michelangela Barbieri, Angela Nebbioso

**Affiliations:** 1 Department of Advanced Medical and Surgical Sciences, University of Campania “Luigi Vanvitelli”, Naples, Italy; 2 Department of Precision Medicine, University of Campania “Luigi Vanvitelli”, Vico L. De Crecchio 7, Naples, Italy; 3 Program of Medical Epigenetics, Vanvitelli Hospital, Naples, Italy; 4 Biogem, Molecular Biology and Genetics Research Institute, Ariano Irpino, Italy; 5 UniCamillus, International Medical University, Rome, Italy

**Keywords:** Cancer genomics, Cancer genetics, Cancer metabolism

## Abstract

Colorectal cancer (CRC) is a leading cause of cancer-related deaths and obesity is a known risk factor for its development and poor prognosis. Adipose tissue (AT) actively contributes to CRC progression and cachexia. Here, we investigated molecular crosstalk between tumor cells and different visceral AT depots (normal, intra- and peri-tumoral), focusing on metabolic and (epi)genetic alterations. Using WGS analysis, we explored VAT role in CRC progression, demonstrating how its proximity to the tumor impacts metabolic and phenotypic changes. Intra-VAT (within 5 cm of lesion), closest to the tumor, underwent significant metabolic remodeling, characterized by upregulation of markers of the white-brown AT transition (UCP-1, TMEM26), lipid metabolism (PON3) and a reduction in adipocyte turnover (Pref-1, adiponectin). Peri-VAT (within 15 cm) and HVAT (over 15 cm) exhibited progressively fewer alterations, suggesting a gradient effect of tumor on surrounding AT. Intra-VAT displayed increased fibrosis (TGF-β, collagen) and cachexia-related markers (IL-8), and mutations in key oncogenes (KRAS, HLA, MET), highlighting a direct interaction between tumor cells and AT driving CRC progression. Mutations in genes such as KRAS, HLA, and PIK3CA were shared between CRC and its Intra-VAT, indicating potential biomarkers for tumor progression and immune evasion. miRNA analysis revealed upregulation of miR-21 and miR-92a in Intra-VAT, with circulating miR-92a correlating with increased body fat and decreased lean mass in CRC patients, suggesting their involvement in both local metabolic remodeling and systemic changes. Altered PON3 DNA methylation patterns were also observed, correlating with metabolic parameters. Our findings underscore AT’s critical role in the CRC microenvironment as an active player in CRC progression and cachexia. Metabolic and genetic alterations decreased in VAT with increasing distance from the tumor. Intra-VAT may serve as a critical therapeutic target and biomarker for CRC progression, impacting surgical and postoperative strategies. Future studies should focus on targeting tumor-adipose crosstalk to improve treatment outcomes, including experimental validation of the identified genetic alterations and investigation of their functional roles in tumor progression and immune evasion.

## Introduction

Colorectal cancer (CRC) represents the third most common cancer worldwide and the second leading cause of cancer-related death [1]. Its progression involves genetic and epigenetic alterations influenced by factors such as nutrition, pharmacological treatments, and lifestyle [2, 3].

Obesity is one of the major risk factors for both the development and the poor prognosis of CRC [4]. A recent study has highlighted the multiple mechanisms by which the adipose tissue (AT) contributes to tumorigenesis [5]. Obesity-induced AT inflammation is marked by elevated circulating pro-inflammatory cytokines and adipokines [6, 7], such as TNF-α, IL-6, IL-8, MCP-1, and leptin, which activate metabolic pathways in tumor cells, promoting their growth and survival [8].

The relationship between AT and CRC is dynamic and bidirectional. CRC can impact AT, by inducing morpho-structural changes, including adipocyte atrophy, imbalances in lipid turnover, and increased lipolysis [8]. Additionally, there is impaired adipocyte turnover, with downregulation of adipogenic genes like PPAR-γ, c-EBPα, and adiponectin, and upregulation of adipogenesis inhibitors such as Pref-1 [8, 9]. Lipid metabolism plays a pivotal role in this context, as alterations in lipid storage and mobilization within AT affect the availability of fatty acids and lipid-derived signaling molecules that are crucial for CRC cell energy supply and proliferation. The reprogramming of lipid metabolism in the tumor-adipose microenvironment facilitates tumor progression by providing essential substrates for membrane synthesis, energy production, and modulation of oncogenic signaling pathways [10, 11]. Moreover, CRC-induced changes in AT promote the release of pro-inflammatory lipid mediators, which further sustain chronic inflammation and immune evasion within the tumor microenvironment [12, 13]. These changes are associated with a browning phenotype, marked by increased uncoupling protein 1 (UCP-1) expression, which enhances thermogenesis, lipid mobilization, and energy expenditure [14, 15]. Furthermore, modifications in the extracellular matrix (ECM) lead to increased expression of fibrosis markers like TGF-β and collagen, contributing to a persistent negative energy balance, inflammation, tumor metabolism, and systemic metabolic alterations [16–18].

Epigenetic deregulation plays a critical role in metabolic reprogramming and tumorigenesis [19]. Specific microRNAs (miRNAs) derived from AT act as regulators, influencing adipogenesis, lipid metabolism, and the tumor microenvironment. These miRNAs also play a critical role in obesity-related inflammation, insulin resistance, cancer progression, and cancer-associated cachexia (CAC) [20–25].

Alterations in DNA methylation of obesity-associated genes have also been observed, reflecting metabolic changes during weight loss. For example, PON3 gene methylation correlates with BMI reduction and could be a biomarker for weight-loss interventions [26–28].

This study investigates the complex crosstalk between AT and CRC by analyzing the metabolic alterations (lipolysis, AT turnover, WAT browning, and fibrosis) in visceral AT (VAT) depots (normal, intra- and peri-tumoral) located at varying distances from the CRC mass. We also explore epigenetic factors and mutations in CRC and VAT within the tumor microenvironment to identify shared genetic pathways that influence both metabolic reprogramming and CRC progression. Additionally, miRNAs implicated in CRC and CAC as well as PON3 DNA methylation were analyzed in peripheral blood mononuclear cells (PBMCs). These markers were correlated with metabolic and nutritional parameters in CRC patients.

## Results

### Clinical characteristics of study population

102 CRC patients and 48 controls were enrolled; their clinical characteristics are summarized in Table 1. CRC patients showed lower fat and muscle mass in percentage and Kg. No differences in fat-free mass (FFM), body cell mass (BCM), or BCMI (body cell mass index) were observed between CRC patients and CTRL (Table 1).

**Table 1:** Main clinical characteristics and anthropometric, metabolic-nutritional parameters, of the study population, stratified into CRC patients and healthy controls (CTRL).

	CTRL (N = 48)	CRC (N = 102)	P value
Age, years	56.9 ± 12.1	66.5 ± 12.2	0.000
Man, n (%)	27 (56.3)	62 (60.8)	0.702
Hypertension, n (%)	16 (33.3)	30 (29.4)	0.927
Diabetes Mellitus, n (%)	10 (20.8)	20 (19.6)	0.861
Hypercholesterolemia, n (%)	16 (33.3)	30 (29.4)	0.339
Obesity, n (%)	13 (27.1)	16 (15.7)	0.1
Other tumors (other locations), n (%)	4 (8.3)	6 (5.9)	0.760
Cardiovascular diseases, n (%)	1 (10)	12 (11.8)	0.452
**Laboratory tests**			
RBC, mil/mm^3^	4.7 ± 0.9	4.2 ± 0.7	0.011
Hb, g/dL	13.3 ± 2.0	12.9 ± 2.3	0.412
WBC, 10^3^/mm^3^	7.1 ± 2.8	8 ± 3.7	0.193
Lymphocytes, N°/mm^3^	2137 ± 698	2011 ± 1408	0.682
Neutrophils, N°/mm^3^	4170 ± 2677	5937 ± 7969	0.297
PLT, N°/µl	267794 ± 127457	249391 ± 113592	0.454
Fibrinogen	407.1 ± 92.1	366.4 ± 185	0.469
Albumin, g/dL	4.2 ± 0.5	3.6 ± 0.6	0.000
Triglycerides, mg/dL	112.1 ± 37.6	127.6 ± 44.7	0.230
Total cholesterol, mg/dL	171.1 ± 40.9	184.2 ± 37.1	0.280
LDL, mg/dL	102.9 ± 36.6	120.1 ± 30.5	0.143
HDL, mg/dL	51.6 ± 14.4	44.2 ± 9	0.073
Glycemia, mg/L	93.6 ± 23.2	108.8 ± 26.5	0.06
Urea, mg/dL	36.9 ± 9.1	39.7 ± 20.4	0.586
Creatinine, mg/dL	0.91 ± 0.33	0.85 ± 0.23	0.272
LDH	179.9 ± 34.5	188.4 ± 53.1	0.632
CRP, mg/dL	1.25 ± 2.05	6.9 ± 8.8	0.003
**Anthropometric parameters**			
BMI, Kg/m^2^	28.1 ± 5.4	26.4 ± 3.9	0.062
WHR	0.93 ± 0.12	0.94 ± 0.09	0.724
Fat free mass, Kg	58.4 ± 10.6	58.1 ± 12	0.917
Fat free mass, %	73.3 ± 10	78.1 ± 12.3	0.078
Fat Mass, Kg	20.8 ± 8.9	15.7 ± 9.8	0.022
Fat Mass, %	25.5 ± 8.5	20.7 ± 11.4	0.044
Muscle mass, Kg	27.4 ± 9.2	28.5 ± 6	0.062
Body cell mass, Kg	32.9 ± 6.7	32.7 ± 9.6	0.620
Body cell mass index	12.9 ± 4.6	11.6 ± 2.8	0.120
**Metabolic-nutritional parameters**			
MEDAS	7.7 ± 2.2	8.7 ± 2.4	0.039
PNI	52.5 ± 5.6	47.2 ± 9.5	0.017
MNA total score	25.6 ± 2.5	23.7 ± 4.2	0.024
NRS screening n (%)	8 (16.7)	36 (35.3)	0.019
**Physical performance**			
SPPB balance test	3.9 ± 0.5	3.6 ± 1	0.213
SPPB total score	9.2 ± 2.6	7.8 ± 3.1	0.021

Values are mean ± SD.

p < 0.05 vs healthy controls (CTRL). MEDAS, Mediterranean Diet Adherence Screener; PNI, prognostic nutrition index; MNA, Mini Nutritional Assessment; NRS, Nutritional Risk score; SPPB, Short Physical Performance Battery.

Regarding metabolic and nutritional parameters, CRC patients had significantly lower Prognostic Nutritional Index (PNI), Mini Nutritional Assessment (MNA) and Mediterranean adherence diet screener (MEDAS) scores, and a significantly higher value of Nutritional Risk Score (NRS) compared to the CTRL (Table 1).

CRC patients also had a significantly lower Short Physical Performance Battery (SPPB) total score, an established measure of physical function and mobility in older adults, indicating impaired physical performance in this group (Table 1).

### Characterization of metabolic derangement of VAT according to tumor distance

#### Tissue biomarkers of metabolic remodeling

The expression levels of UCP-1 and TMEM26, markers of the WAT-to-BAT transition, and PON3, a marker of lipid metabolism, were evaluated to characterize VAT’s metabolic derangement according to tumor distance.

**Fig. 1:**
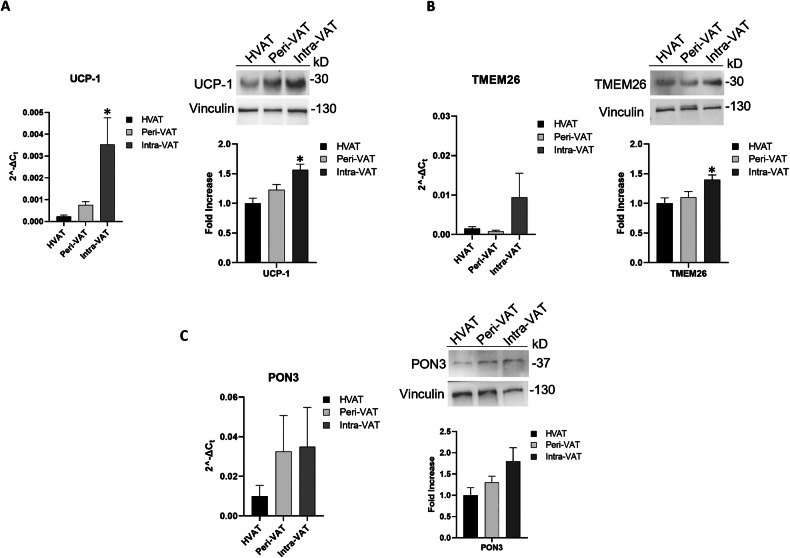
Detection of tissue biomarkers of metabolic remodeling in AT from CRC patients. mRNA expression levels of (**A**) UCP-1, (**B**) TMEM26, (**C**) PON3 in visceral adipose tissue (HVAT), peritumoral-visceral adipose tissue (Peri-VAT) and intra-tumoral-visceral adipose tissue (Intra-VAT) from CRC patients (left panels). The fold increase of mRNA expression was calculated using the 2^−ΔCt^ method; actin was used as internal control. Data are mean ± standard errors. *p < 0.05 vs HVAT samples. (*n* = 10 for each group). **A**, **B**, **C** Western blot analysis for adipose UCP-1, TMEM26, PON3 (right panels). The histograms show the densitometric analysis. HVAT protein expression was set as 1 and values are expressed as fold increase. Data are mean ± standard errors. *p < 0.05 vs HVAT samples.

Gene and protein expression of UCP-1 showed a progressive increase among the three AT groups, reaching the highest level in Intra-VAT samples (*p < 0.05) (Fig. 1A). This is consistent with previous evidence supporting UCP-1 as a key regulator of thermogenesis and metabolic remodeling in AT [29, 30]. In addition, the expression of TMEM26 was significantly increased in Intra-VAT samples compared to HVAT samples (*p < 0.05) (Fig. 1B), corroborating its role as a marker of beige adipocyte differentiation [31, 32].

PON3 gene and protein expression levels showed an increasing trend from HVAT to Intra-VAT, although these differences did not reach statistical significance (Fig. 1C). This finding aligns with studies highlighting PON3 involvement in oxidative lipid metabolism and protection against oxidative stress [33, 34].

#### Tissue biomarkers of AT turnover

AT turnover was evaluated by analyzing the adiponectin/Pref1 ratio and the expression levels of Caspase-3 to investigate the metabolic derangement of VAT based on tumor distance.

**Fig. 2:**
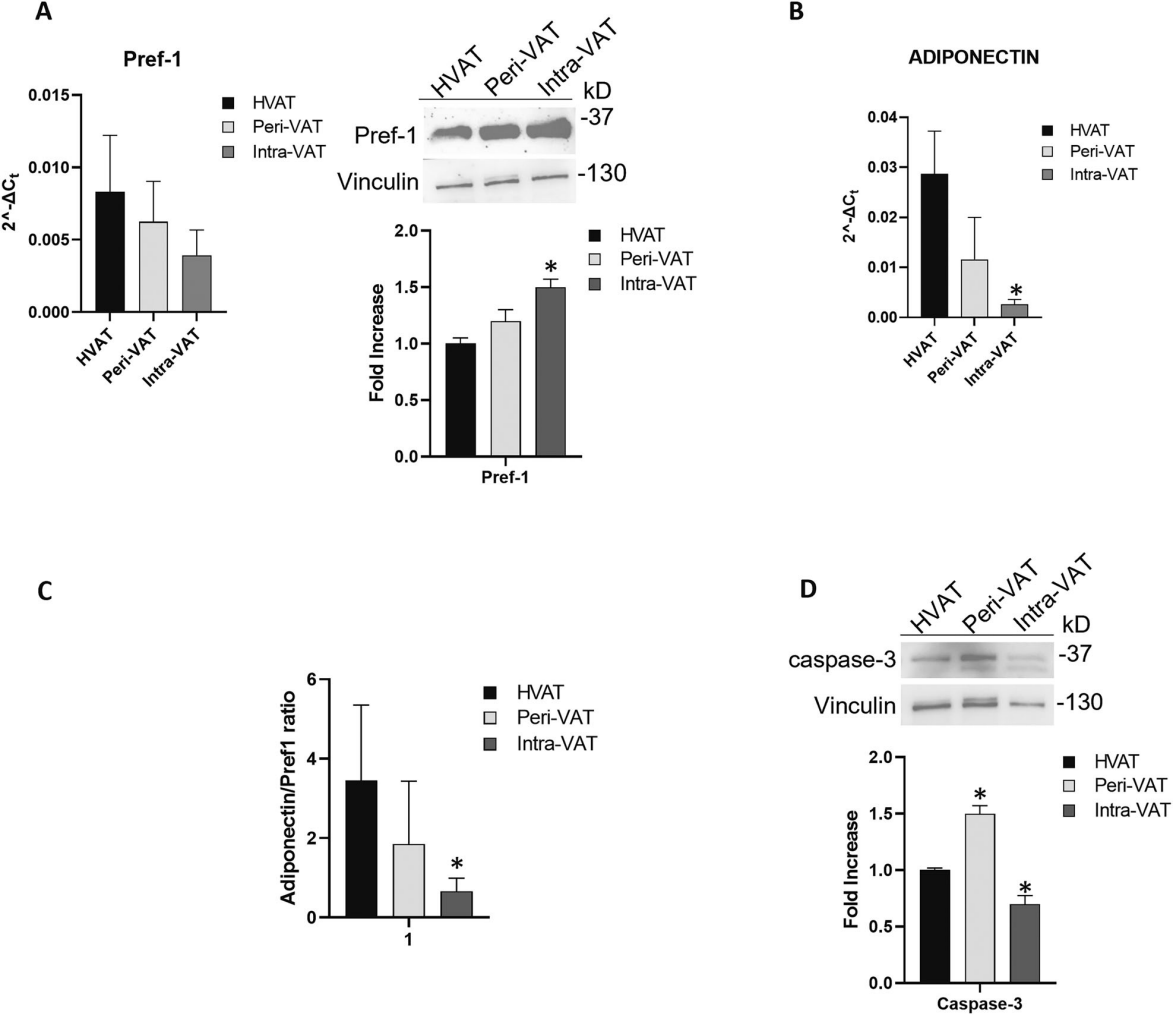
Detection of tissue biomarkers of AT turnover in ATs from CRC patients. **A**, **B** AT relative mRNA levels of Pref-1 and Adiponectin. The fold increase of mRNA expression was calculated using the 2^−ΔCt^ method; actin was used as internal control. (*n* = 10 for each group). **A**, **D** Western blot analysis for adipose Pref-1 and Caspase-3. The histograms show the densitometric analysis. HVAT protein expression level was set as 1 and values are expressed as fold increase. Vinculin was used as internal control. Data are mean ± standard errors. *p < 0.05 vs HVAT samples. **C** Adiponectin/Pref-1 ratio. The ratio was calculated from mRNA expression levels of each group.

Gene expression of pre-adipocyte factor 1 (Pref-1), a marker of pre-adipocytes, showed a slight decrease across the three groups of AT (Fig. 2A, left panel). In contrast, its protein expression was significantly higher in Peri-VAT and Intra-VAT compared to HVAT samples (*p < 0.05) (Fig. 2A, right panel). This discrepancy suggests post-transcriptional regulation mechanisms that modulate protein levels independently of mRNA expression, a phenomenon commonly observed in AT biology [35, 36]. In particular, the increase in Pref-1 protein despite reduced mRNA in Intra-VAT may result from enhanced protein stability or translation efficiency, altered degradation pathways, or the influence of the tumor microenvironment, as reported in pathological contexts such as cancer [37–39]. Adiponectin gene expression demonstrated a progressive decrease among the three AT groups, with the lowest expression level observed in Intra-VAT samples (*p < 0.05) (Fig. 2B). The adiponectin/Pref1 ratio, an established marker of AT turnover, also decreased progressively, reaching its lowest value in Intra-VAT samples (*p < 0.05) (Fig. 2C), in line with previous reports linking decreased adiponectin to impaired adipocyte function in cancer cachexia [16, 40].

Protein expression of caspase 3, a marker of apoptosis and cellular turnover in AT, was significantly reduced in Intra-VAT samples (*p < 0.05) (Fig. 2D), consistent with altered apoptotic dynamics in tumor-associated AT [41, 42].

#### Tissue biomarkers of cancer cachexia and fibrosis

VAT’s metabolic derangement according to its proximity to the tumor was further characterized by analyzing markers of CAC (IL-8, IL-10, and IL-15) and fibrosis (collagen, TGF-β).

**Fig. 3:**
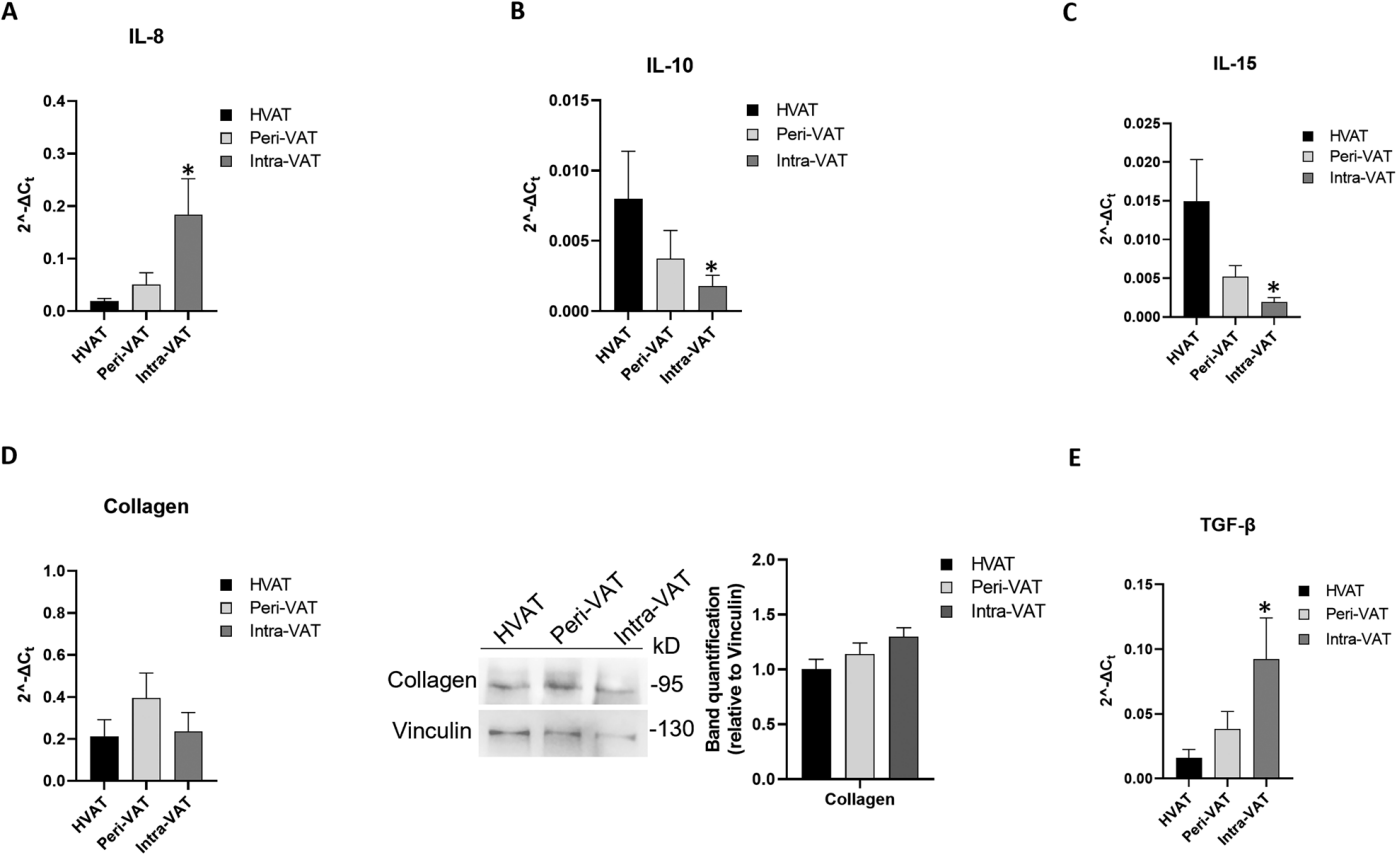
Detection of tissue biomarkers of cachexia and fibrosis in AT from CRC patients. AT relative mRNA levels of (**A**) IL-8, (**B**) IL-10, (**C**) IL-15, (**D**) collagen (left panel), (**E**) TGF-β. The fold increase of mRNA expression was calculated using the 2^−ΔCt^ method; actin was used as internal control. Data are mean ± standard errors. *p < 0.05 vs HVAT samples. (*n* = 10 for each group). **D**, right panel). Western blot analysis for adipose collagen. The histograms show the densitometric analysis. Values are expressed as fold increase. Vinculin was used as internal control. Data are mean ± standard errors. *p < 0.05 vs HVAT samples.

Gene expression of the pro-CAC marker IL-8 was significantly elevated in Intra-VAT compared to HVAT samples (*p < 0.05) (Fig. 3A). Gene expression of the anti-CAC markers, IL-10 and IL-15, progressively decreased between the AT groups, with the lowest expression levels in Intra-VAT (*p < 0.05) (Fig. 3B, C).

For the fibrosis markers, both gene and protein expression of collagen were increased in Peri-VAT and Intra-VAT compared to HVAT samples, although the differences did not reach statistical significance (Fig. 3D). This may reflect biological variability and the complexity of extracellular matrix remodeling, involving multiple ECM components beyond type I collagen [43, 44], and regulation that depends on disease stage or post-transcriptional mechanisms [45].

However, the expression of TGF-β showed a progressive increase, reaching the highest expression level in Intra-VAT (*p < 0.05) (Fig. 3E).

### Genetic alterations in AT in CRC patients contributing to tumorigenesis and immune escape

**Fig. 4:**
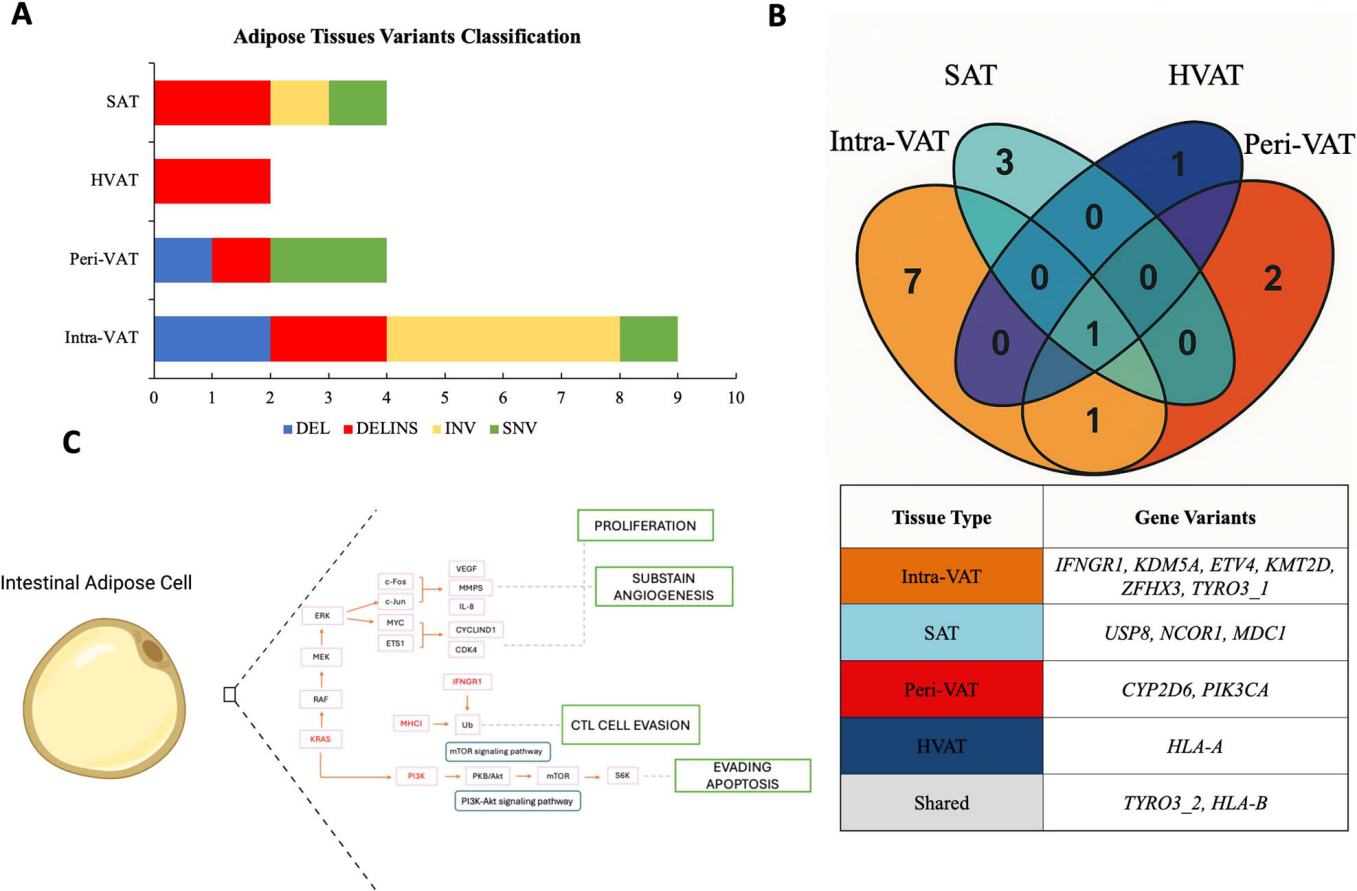
Genetic Variant Classification and Distribution in ATs Associated with CRC. **A** *Classification* of *AT Variants:* Bar graph displaying the distribution of genetic variants type, deletions (DEL), insertion-deletions (DELINS), inversions (INV), and single nucleotide variants (SNVs), identified in AT adjacent to CRC, including Intra-VAT, Peri-VAT, HVAT and SAT. Intra-VAT group shows the highest diversity, especially in SNVs. **B** *Shared and Exclusive Variants in ATs*: (Up) Venn diagram illustrating the overlap and exclusivity of genetic variants among the four AT types. Intra-VAT harbors the highest number of unique variants (*n* = 7), while HLA-B and TYRO3_2 represents the only variants shared across all tissue types; (Down) related panel detailing the specific shared and exclusive variants. **C** *Key signaling pathways involving mutated genes in adipose cells within and surrounding CRC tissue*: Schematic representation of key signaling pathways affected by genes mutated in adipose cells located within or adjacent to CRC tissues. These pathways are implicated in cancer-related processes such as cell proliferation, immune evasion, and angiogenesis. (*n* = 3 per group). Panel C was created using BioRender (https://biorender.com/).

Analysis of the presence of genetic variants across all four AT types revealed that the frequency of gene mutations varied between tissues, with Intra-VAT showing the highest incidence of genetic variants (Fig. 4A). This finding suggests a greater degree of DNA damage in the AT located within the tumor bulk, as a result of cellular communication or material exchange with malignant cells. A Venn diagram in Fig. 4B summarizes the unique and shared genetic variants across the different AT types. Specifically, the Intra-VAT-only region (orange) contained seven unique variants in the genes IFNGR1, KDM5A, TYRO3_[1], KMT2D, ZFHX3 and ETV4 with two variants. The SAT-only region (cyan) showed three unique variants in the genes USP8, NCOR1, and MDC1. The Peri-VAT-only region (red) displayed two unique variants in the genes CYP2D6 and PIK3CA and the HVAT-only region (blue) was associated with a single variant in the HLA-A gene. Some genetic variants were shared between tissue types (ie the TYRO3 [2] and the HLA-B variant).

Functional pathways analysis of these mutations (Fig. 4C) highlighted their involvement in key cancer-related processes, including proliferation and angiogenesis (KRAS), cytotoxic T lymphocyte (CTL) evasion (HLA-A, HLA-B, HLA-C, IFNGR1), and apoptosis evasion (KRAS, PI3K).

Table 2 details the identified genetic alterations and lists the specific mutation types. Several studies have demonstrated a link between these variants and mechanisms that promote uncontrolled cell growth [46–51]. Notably, some of these genes, including IFNGR1, HLA-C, and HLA-B, are involved in immune response. When mutated, they can lead to immune escape, an important cancer hallmark [52].

**Table 2:** Gene Variants Detected in ATs.

Tissue type	Gene Name	cDNA Variant
Intra-VAT	IFNGR1	NM_000416.3:c.1251_1254inv
Intra-VAT	KDM5A	NM_001042603.3:c.2_13delinsC
Intra-VAT	ETV4_(1)	NM_001079675.5:c.604 C > T
Intra-VAT	ETV4_(2)	NM_001079675.5:c.609_613inv
Intra-VAT	KMT2D	NM_003482.4:c.2382_2393inv
Intra-VAT	TYRO3_(1)	NM_006293.4:c.2145+1_2145+211del
Intra-VAT	ZFHX3	NM_006885.4:c.10520_10529inv
SAT	USP8	NM_005154.5:c.2353 A > G
SAT	NCOR1	NM_006311.4:c.5685_5702inv
SAT	MDC1	NM_014641.3:c.3528_3529delinsAT
HVAT	HLA-A	NM_002116.8:c.964_967delinsTTCG
Peri-VAT	CYP2D6	NM_000106.6:c.886 C > T
Peri-VAT	PIK3CA	NM_006218.4:c.1624 G > C
Intra-VAT + Peri-VAT	TYRO3_(2)	NM_006293.4:c.1483+1_1483+263del
Intra-VAT + Peri-VAT + HVAT + SAT	HLA-B	NM_005514.8:c.311_319delinsTCGCGCTCC

The table lists the gene names and cDNA variants detected in the different AT samples. Notable findings include variants in *TYRO3*, *ETV4*, *KDM5A*, and *HLA-B*, which are either specific to or shared across multiple tissue types. The *HLA-B* variant is shared across all AT types, suggesting its potential role in CRC-AT cross-talk.

### Selected CRCT mutations driving gene silencing in tumor progression

**Fig. 5:**
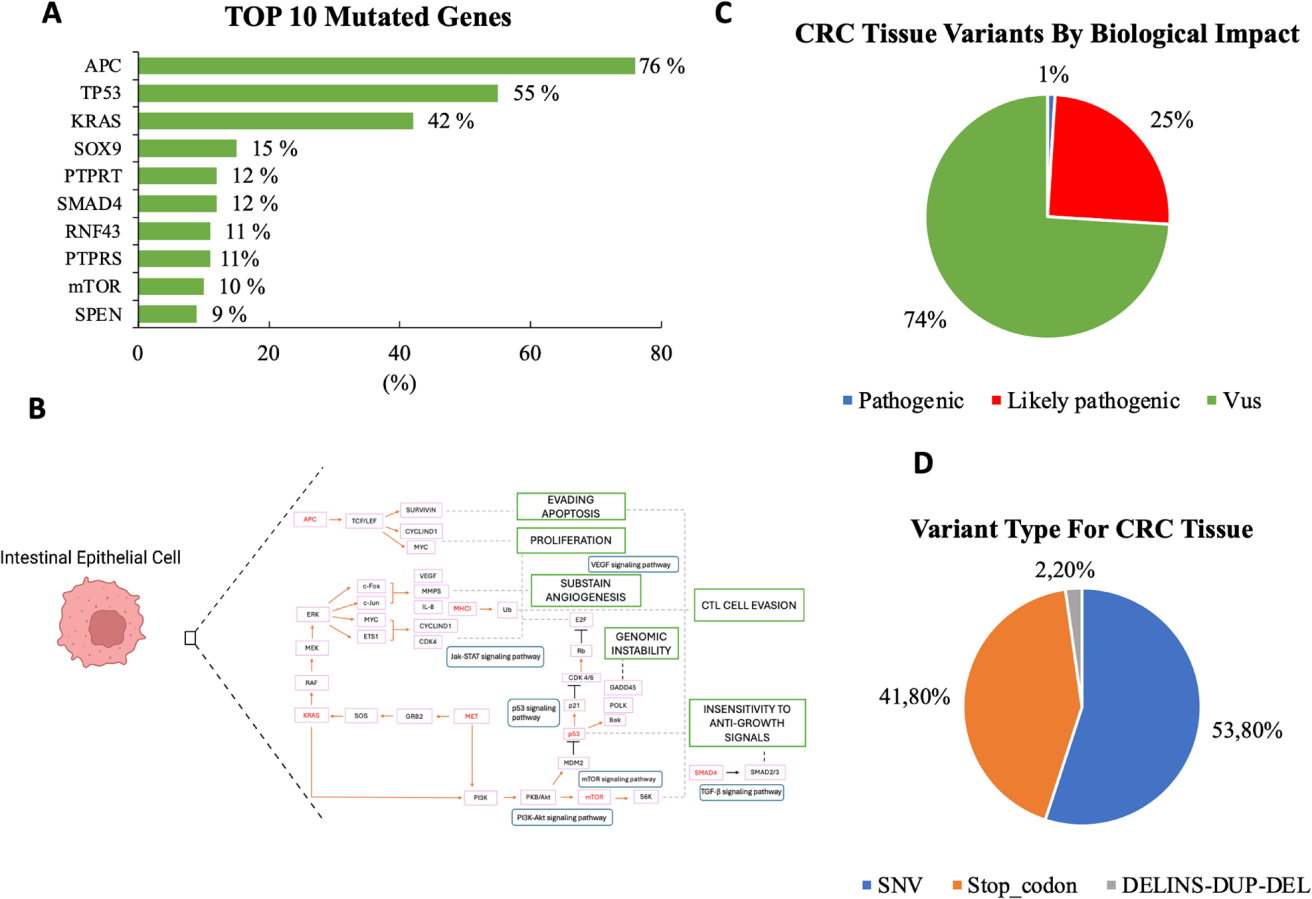
Genomic landscape of CRC variants. **A** *Top 10 Mutated Genes in CRC*: Bar graph showing the top 10 most frequently mutated genes in CRC, with *APC* (76%), *TP53* (55%), and *KRAS* (42%) as the most common. **B** *Pathways involvement of frequently mutated genes*: Schematic illustration of the top 10 most frequently mutated CRC genes (shown in red) and their involvement in key signaling pathways. **C** *Biological Impact of CRC Variants*: Pie chart illustrating the functional classification of detected variants in CRC tissues. Variants of uncertain significance (VUS) represented the majority (74%), while pathogenic and likely pathogenic variants accounted for 1% and 25%, respectively. **D** *Distribution of Variant Type in CRC Tissue*: Pie chart showing the relative proportion of mutation types. Single nucleotide variants (SNVs) were the most prevalent (53.8%), followed by stop codon mutations (41.8%) and a minor fraction of insertion/deletion events (2.2%). Panel B was created using BioRender (https://biorender.com/).

Genetic analysis of selected CRC tissue (CRCT) samples showed overlapping genetic mutations, supporting the well-established hypothesis that common mutations drive and sustain carcinogenesis [53]. Among the analyzed tumoral samples, the top 10 most frequently mutated genes are shown in Fig. 5A. Specifically, pathways associated with proliferation and apoptosis evasion (APC, mTOR), genomic instability (TP53), angiogenesis (KRAS, MET) and immune evasion (HLA-A, HLA-B, HLA-C) were prominently affected. Mutations in critical genes such as APC, KRAS, and TP53 disrupt normal cellular processes, promoting tumor growth, survival, and resistance to anti-proliferative signals. Similarly, mutations in SMAD4, a component to the TGF-β pathway, further contribute to CRC progression by impairing tumor suppressive functions (Fig. 5B).

Further investigation of the common genetic mutations revealed that they were primarily composed of stop codon (nonsense) mutations (41.8%) and missense mutations (53.8%), with a smaller percentage classified as likely pathogenic (25%) or pathogenic (1%) variants (Fig. 5C, D).

To validate these sequencing results, two genes—APC and KRAS—were selected for additional analysis using RT-qPCR with primers targeting sequences both before and after the mutation site (Supplementary Table 1A, B). RT-qPCR analysis showed no detectable mRNA expression for the 4314_4315dup APC mutation when primers targeting sequences downstream of the mutation site were used (Supplementary Fig. 1A). Similarly, no mRNA expression was observed for the 38 G > A missense mutation in KRAS when primers targeting sequences upstream of the mutation site were utilized (Supplementary Fig. 1B).

Taken together, the findings suggest that specific mutations in CRCT not only drive malignant growth but may also lead to transcriptional silencing of the affected genes, further contributing to tumor progression.

### Common mutations in CRC and AT promoting tumor growth and immune evasion

To further explore CRC-AT crosstalk, an in-depth analysis was conducted to identify genetic mutations common to both CRC and AT, as summarized in Table 3. These shared mutations may facilitate bidirectional communication between CRC and AT, contributing to a microenvironment that supports cell proliferation and survival. Such crosstalk could occur through the modulation of molecular signals and growth factors secreted by AT, which may influence local metabolism and immune responses, with potential implications for the development of novel therapeutic strategies and the prognosis of CRC.

**Table 3:** Gene variants detected in CRC and AT.

Tissue type	Gene Name	cDNA Variant
CRC + Intra-VAT	HLA-C	NM_002117.6:c.538_539delinsTG
CRC + Peri-VAT	KRAS	NM_033360.4:c.38 G > A
CRC + SAT	CD79A	NM_001783.4:c.87_89inv
CRC + Intra-VAT + Peri-VAT	MET	NM_000245.4:c.3029-721dup
CRC + Intra-VAT + SAT	HLA-B	NM_005514.8:c.353_355delinsTCA
CRC + Peri-VAT + SAT	CD79A	NM_001783.4:c.95 C > G
CRC + Intra-VAT + SAT + HVAT	HLA-A	NM_002116.8:c.257_259delinsGGC

The table lists the gene names and cDNA variants detected in different samples of CRC and AT. Notable findings include variants in HLA-C, KRAS and CD79A, specific between CRC and various types of AT. MET, HLA-B, CD79A variants are shared between CRC and two types of AT. HLA-A variant is shared between CRC and three types of AT.

**Fig. 6:**
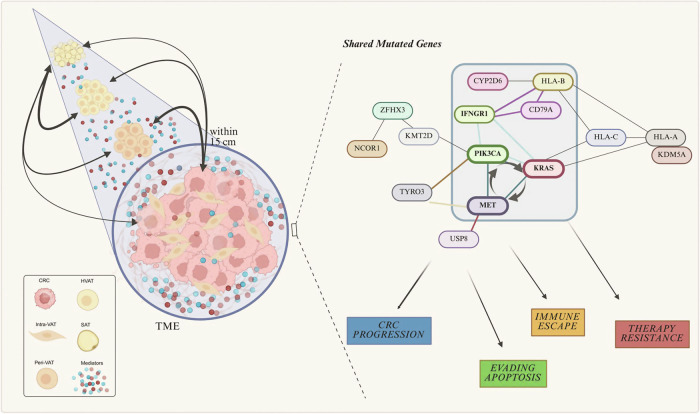
Molecular pathways mediated by shared mutations in CRC and AT cells. Schematic representation of the tumor microenvironment (TME) illustrating the molecular interplay between CRC cells and surrounding AT compartments, including Intra-VAT, Peri-VAT, HVAT, and SAT. Shared mutations in genes are implicated in the activation of key oncogenic processes, including tumor progression, immune evasion, resistance to apoptosis, and therapy resistance. This model highlights potential crosstalk mechanisms between CRC and AT. This figure was created using BioRender (https://biorender.com/).

Figure 6 illustrates the signaling pathways influenced by shared mutations between CRCT and AT. For example, mutations like KRAS in epithelial cells drive pathways such as ERK/MEK and PI3K-Akt, promoting key oncogenic processes, including proliferation, evasion of apoptosis, angiogenesis, and resistance to anti-growth signals (BCR, e.g., CD79A). Additionally, CTL response evasion is associated with mutations in major histocompatibility complex class I (MHCI). Adipose cells contribute to the tumor microenvironment by influencing these processes through cytokine and growth factor signaling, thereby supporting cancer cell survival and immune evasion. These interconnected signaling pathways further reinforce the interactions between CRC and AT.

Mutations in genes such as HLA-C, KRAS, CD79A, MET, HLA-B, and HLA-A were found to be simultaneously mutated in both AT and CRCT. AT releases pro-inflammatory adipokines and cytokines, which can activate the PI3K-Akt pathway in CRC tumor cells [54]. Mutations in KRAS gene activate this pathway, promoting survival and proliferation of cancer cells [55]. Once AT-derived growth factors activate, the MET gene, which encodes a tyrosine kinase receptor, may stimulate the PI3K-Akt pathway, promoting tumor growth and chemoresistance [56]. MET is also involved in anti-apoptotic signaling, potentially contributing to increased resistance to apoptosis in CRC cells [57, 58]. KRAS mutations are among the most frequently recurrent in CRC and cooperate with other signaling genes, such as K-Ras, Raf, MEK and ERK, in the activation of the MAPK pathway which adipokines produced by AT can also modulate. Adipokines interact with CRC cell receptors, amplifying proliferation signals through the MAPK pathway [59, 60]. Mutations in KRAS thus promote an environment where AT contributes to the growth and resistance of CRC cells.

HLA-A, HLA-B, and HLA-C genes play a key role in the regulation of the immune response. Mutations in these genes are able to alter the immune system’s capability to recognize and destroy cancer cells [61]. In particular, CRC cells expressing these mutations may escape immune control, creating an environment conducive to their survival. AT, on the other hand, may help modulate the immune response by producing pro-inflammatory cytokines.

### Common HLA mutations between CRC and AT could have emphasized the role of crosstalk in the creation of an immunosuppressive microenvironment supporting tumor growth

CD79A interacts with B cells, playing a role in the immune system, and its mutation could allow CRC cells to escape the immune response [62, 63]. This condition, together to stimuli from AT, may create an environment in which CRC cells can grow uncontrollably.

Furthermore, the data analysis revealed that some genes exhibit many mutations, even if classified as benign or likely benign. However, these mutations could still contribute to the process of carcinogenesis [64]. The set of mutations reported in Table 3 and detected both in CRC and AT could have a determining role in initiating the neoplasia, once again highlighting potential crosstalk between CRC and AT. This molecular dialogue between the two tissues may have influenced the onset of the tumor, underscoring the complexity of the interactions between CRC and AT.

### miR-21 and miR-92a upregulation in AT suggests a potential involvement in CRC progression

**Fig. 7:**
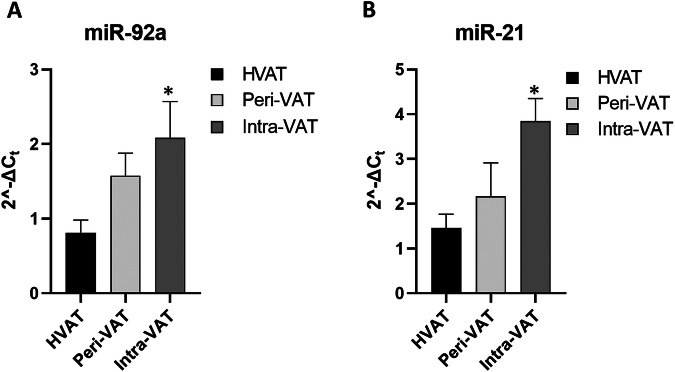
miR-92a and miR-21 expression levels in ATs at different distances from tumor mass in CRC patients. VAT relative expression of **A** miR-92a and **B** miR-21 in CRC patients. The fold increase of miRNA expression was calculated using the 2^−ΔCt^ method. Let-7-g was used as internal control. Data are mean ± Standard Errors. *p < 0.05 vs. HVAT samples.

Given the critical role of epigenetic alterations in cancer progression and cachexia, the expression level of a panel of miRNA [65] was also evaluated in ATs at different distances from tumor mass in CRC patients. Cachexia is characterized by weight loss, muscle wasting, and fat depletion, which are often observed in patients with advanced cancer, particularly in CRC. Several miRNAs have been implicated in regulating key processes related to cancer cachexia, such as inflammation, metabolism, and tissue remodeling. The panel included the following miRNA: miR-21, miR-92a, miR-181b, miR-203a, miR-23a, and miR-1246. Among these, miR-92a and miR-21 were the most highly expressed. The expression of miR-92a showed a progressive increase from HVAT to Intra-VAT samples (*p < 0.05) (Fig. 7A), while miR-21 expression was significantly increased in Intra-VAT samples compared to HVAT samples (*p < 0.05) (Fig. 7B).

These results indicate a significant upregulation of miR-21 and miR-92a in ATs located in proximity of tumor tissue, suggesting their potential involvement in the cancer microenvironment and tumor progression.

### Expression levels of miR-92a and miR-21 in circulating PBMC

The expression levels of the panel of miRNA analyzed in VAT were also evaluated in circulating PBMCs from the entire patient cohort to assess whether circulating leukocytes could reflect the AT-specific epigenetic profile.

**Fig. 8:**
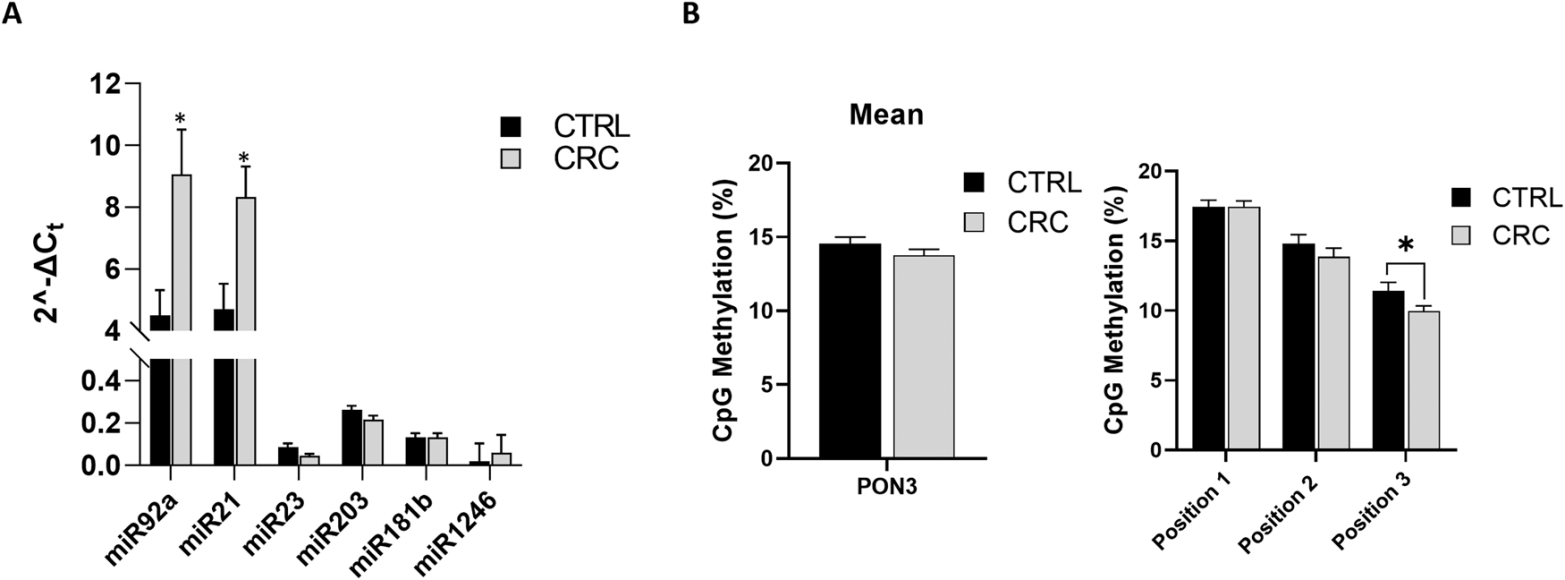
Circulating epigenetic markers in PBMCs from CRC patients and control group (CTRL). **A** Expression of a panel of miRNA in PBMCs from CTRL and CRC subjects. The histograms show 2^^-ΔCt^ levels of differentially expressed miRNAs. Data are mean ± Standard Errors; *p < 0.05 vs CTRL. **B** DNA methylation analysis of PON3 in PBMC samples from CTRL and CRC subjects. The DNA methylation status is expressed as percentage of CpG methylation. Data are mean ± Standard Errors. *p < 0.05 vs. CTRL subjects. (*n* = 48 CTRL and *n* = 102 CRC).

PBMCs isolated from CRC patients exhibited higher expression levels of miR-92a and miR-21 compared to PBMCs from healthy controls (*p < 0.05) (Fig. 8A). Interestingly, a direct correlation between miR-92a and miR-21 expression levels was observed (r = 0.203, p = 0.031).

Correlation analysis of anthropometric and metabolic parameters with miR-92a and miR-21 expression levels in PBMC was also performed. In the entire study population, miR-92a expression levels were positively correlated with body fat percentage (r = 0.247, p = 0.043) and negatively correlated with FFM (r = -0.288, p = 0.013), skeletal muscle mass (r = -0.262, p = 0.031), BCM (r = -0.346, p = 0.005) and BCMI (r = -0.256, p = 0.048). miR-21 expression levels showed a trend of negative correlation with body fat, BCM and BCMI, although these correlations were not significant. (Supplementary Table 2A).

### PON3 methylation status in circulating PBMC

Given the different expression of PON3 in ATs based on the distance from the tumor and its role in metabolism, PON3 DNA methylation was also evaluated at circulating levels to identify potential epigenetic biomarkers associated with metabolic derangements and cachexia in CRC patients.

PON3 DNA methylation, associated with the metabolic remodeling, was also evaluated in PBMC samples. While no statistically significant differences in mean PON3 DNA methylation were observed between CRC patients and healthy controls (CTRL), the analysis of individual positions within the PON3 gene revealed that methylation was significantly decreased at position 3 of the gene promoter in CRC patients (*p < 0.05) (Fig. 8B).

The mean PON3 DNA methylation levels were positively correlated with FFM (r = 0.318, p = 0.009) and BCM (r = 0.245, p = 0.048), and negatively correlated with body fat percentage (r = -0.320, p = 0.008) and WHR (r = -0.371, p = 0.007). A significant correlation was found between the DNA methylation of position 3 of the PON3 promoter and MNA score (r = 0.251, p = 0.031).

Interestingly, a significant inverse correlation between the methylation of position 3 of PON3 gene promoter with miR-92a expression levels was also observed (r = -0.268, p = 0.006).

Moreover, a direct correlation between mean PON3 DNA methylation levels and the Karnofski index, which indicates patients’ performance status, was also found (r = 0.453, p = 0.022) (Supplementary Table 2B).

These results suggest that CRC patients exhibit higher levels of circulating miR-21 and miR-92a, with miR-92a positively correlated with body fat percentage and inversely correlated with FFM, BCM and BCMI. While mean circulating PON3 DNA methylation levels did not change between CRC and CTRL, specific decreases at position 3 of the PON3 promoter were associated with altered metabolic and nutritional parameters and negatively correlated with miR-92a expression.

## Discussion

Our study highlights the critical role of VAT in CRC progression, revealing distinct phenotypic changes that depend on the tumor’s distance from the tumor. VAT closest to the tumor displayed a significantly altered metabolic phenotype, including reduced expression of markers associated with AT turnover and increased expression of markers associated with WAT browning, CAC, and fibrosis. These biomarkers (UCP-1, TMEM26, PON3, Caspase-3, adiponectin/Pref-1) were selected based on robust literature evidence to explore key aspects of AT remodeling and its interaction with CRC. The novelty of our study lies in spatially assessing their expression relative to tumor proximity (HVAT, Peri-VAT, Intra-VAT), providing a comprehensive metabolic and molecular characterization of visceral AT in CRC patients. In particular, PON3 expression was elevated in Intra-VAT, suggesting its involvement in metabolic remodeling during weight loss and highlighting its potential as a biomarker for AT dysfunction in CRC [66].

Analysis of miRNA expression revealed upregulation of miR-21 and miR-92a in Intra-VAT, along with increased levels of UCP-1 and TMEM26, indicative of a WAT-to-BAT transition [25]. These findings underline the influence of the tumor microenvironment on local AT, driving phenotypic changes that may facilitate tumor progression [67]. Elevated circulating levels of miR-21 and miR-92a were also observed in CRC patients, which correlated with anthropometric and metabolic parameters. miR-92a levels were positively correlated with body fat percentage and negatively with FFM, BCM, and BCMI, while miR-21 was associated with BMI and WHR. These correlations suggest a systemic role for these miRNAs in CRC-associated metabolic alterations, linking local AT remodeling with broader systemic effects [68, 69].

Epigenetic analysis revealed reduced DNA methylation at position 3 of the PON3 gene promoter in CRC patients, which was positively correlated with FFM and negatively with body fat percentage and WHR. The inverse correlation between PON3 methylation and circulating miR-92a levels suggests a coordinated regulatory mechanism that links AT metabolism, systemic metabolic parameters, and epigenetic changes in CRC. Furthermore, the positive correlation between PON3 methylation and Karnofsky performance status supports its potential as a marker of metabolic health and disease progression [27].

Additionally, analysis of shared genetic mutations between CRCT and VAT revealed the presence of variants in key genes, including KRAS, MET, CD79A, and HLA, involved in proliferation, immune evasion, and metabolism [70]. The co-existence of these mutations in both compartments suggests a close interaction between the tumor and AT microenvironment. This interaction likely enhances metabolic and inflammatory signaling, contributing to malignancy, with potential implications for personalized therapeutic strategies. Several genetic alterations identified concurrently in CRC and AT compartments may actively contribute to tumor progression and immune evasion by dysregulating key signaling pathways (Fig. 6). Notably, MET mutations were detected in both CRC and Intra-VAT and Peri-VAT. The MET variant identified (NM_000245.4:c.3029-721dup) may lead to dysregulated MET activation, promoting oncogenic processes such as proliferation, invasion, angiogenesis, and metastasis. Importantly, MET functions upstream of major cancer-related signaling cascades, including RAS/MAPK and PI3K/AKT pathways. We also identified a pathogenic KRAS mutation (c.38 G > A, p.G13D) in both CRC and Peri-VAT, along with a PIK3CA variant in Peri-VAT. These mutations are significant as MET directly activates KRAS and PIK3CA downstream effectors, contributing to tumor cell survival, growth, and immune evasion [71, 72]. Co-occurrence of these alterations suggests a cooperative MET–KRAS–PIK3CA oncogenic axis, active not only within CRC but also across Intra-VAT and Peri-VAT compartments.

Notably, these two AT depots harbor two TYRO3 mutations. TYRO3, member of the TAM receptor family, activates the PI3K/AKT pathway and plays a central role in suppressing immune responses by inhibiting dendritic cell activity and promoting tolerogenic signaling. The simultaneous presence of MET and TYRO3 mutations within the tumor-associated adipose niche might foster an immunosuppressive microenvironment, facilitating immune escape and tumor progression.

Additionally, USP8, a deubiquitinase regulating receptor tyrosine kinases such as MET and EGFR, was found mutated exclusively in SAT, potentially further sustaining MET signaling. Indeed, aberrant USP8 function can lead to prolonged activation of these oncogenic receptors, indirectly enhancing MET- and PI3K-mediated signaling even in ATs distant from the tumor bulk. These findings support the presence of an interconnected MET–KRAS–PIK3CA network across tumor and adipose compartments, contributing to CRC progression, immune evasion, and potentially therapy resistance.

A potential link to immune evasion mechanisms is supported by mutations in CD79A detected in CRC, Intra-VAT, and SAT. CD79A is essential for B cell antigen receptor signaling, and its mutation may impair immune surveillance. The presence of CD79A mutations in both CRC and tumor-adjacent ATs suggests these mutations may facilitate an immunosuppressive microenvironment, interfering with effective antitumor immune responses [63].

Furthermore, mutations in IFNGR1, encoding the α-chain of the interferon-γ receptor essential for antitumor immunity via JAK/STAT signaling, were identified exclusively in the Intra-VAT compartment, suggesting localized immune signaling impairment. Notably, KRAS and PIK3CA mutations have also been implicated in suppressing IFN-γ signaling pathways, potentially compounding immune evasion mechanisms. The co-occurrence of these mutations indicates a coordinated mechanism promoting immune escape within CRC and surrounding AT.

Lastly, mutations in HLA class I genes, specifically HLA-B—which is crucial for presenting endogenous antigens to cytotoxic T lymphocytes and is linked to immune evasion in cancer—were identified across CRC and multiple adipose compartments [73, 74]. Additionally, mutations in CYP2D6, involved in xenobiotics and drugs metabolism and implicated in modulating immune responses, were identified specifically in the Peri-VAT [75, 76]. The co-occurrence of these mutations might collectively contribute to immune evasion and altered metabolic functions within AT compartments, highlighting the complex interactions shaping the tumor-adipose microenvironment.

These findings highlight AT as an active participant in CRC progression, extending beyond its traditional role as an energy reservoir. AT remodeling and its reciprocal interactions with CRC cells shape both metabolic and immune dynamics, promoting tumor growth and resistance to therapy [7, 77]. Targeting these interactions presents potential therapeutic opportunities, particularly in the management of cachexia and metabolic dysfunction in CRC. Future studies should focus on unraveling the molecular mechanisms driving this AT-CRC crosstalk, developing strategies to modulate these pathways, and ultimately improving patient outcomes through personalized treatment approaches [78].

Overall, our analysis highlights AT’s adaptive response to the influence of the tumor microenvironment, which may not only facilitate tumor progression but also affect surgical outcomes and clinical follow-up.

A particularly relevant clinical issue concerns the surgical management of VAT close to the tumor. Our data suggest that Intra-VAT, the AT closest to the tumor (within 5 cm from the lesion), exhibits the most significant phenotypic and metabolic alterations, which may justify its removal during surgery [79]. Removing this AT during tumor resection could reduce the risk of local recurrence by eliminating a microenvironment promoting persistent pro-tumor signaling and chronic inflammatory processes [54, 79]. However, the optimal extent of VAT resection remains to be clarified: a too-conservative approach (limited to Intra-VAT) may leave involved tissue behind. In contrast, a more extensive resection (including Peri-VAT) may negatively impact energy balance and postoperative recovery [80].

Another point to consider is the potential use of residual VAT as a useful biomarker for post-operative follow-up [5]. A biopsy of the remaining VAT after surgery may offer valuable insight into potential molecular or epigenetic alterations over time, indicating early recurrence or persistent CRC-related metabolic reprogramming [27, 55, 68]. This minimally invasive approach could complement current cancer surveillance strategies, improving the ability to detect subclinical recurrences or changes in the intestinal microenvironment over time after CRC resection [68, 78]. Furthermore, longitudinal monitoring of miR-92a and miR-21 expression levels via liquid biopsy after surgical resection could provide important information to detect early modifications associated with tumor recurrence or silent disease progression.

In conclusion, our study highlights the importance of an integrated approach to CRC management, considering the tumor and surrounding AT as a key player in disease progression and monitoring [68]. The analysis of VAT and its molecular and epigenetic alterations opens new perspectives for oncological surgery: the definition of the optimal resection margins and the use of VAT biopsies could improve diagnostic accuracy and early detection of recurrences [55, 68, 78]. Future studies should investigate the relationship between the AT microenvironment and CRC further, exploring targeted interventions to modulate AT metabolism and evaluating the clinical utility of epigenetic monitoring in the follow-up of surgically treated patients [68, 78].

## Materials and methods

### Study population enrollment

A total of 102 patients with colorectal cancer (CRC) and 48 healthy controls (CTRL) were enrolled at the Unit of General and Geriatric Surgery, University of Campania ‘L. Vanvitelli’. The study included patients diagnosed with colon carcinoma, as well as rectal and recto-sigmoid cancers. Exclusion criteria were applied to ensure a homogeneous study population. Patients with previous or ongoing anticancer or continuous anti-inflammatory treatments, liver or renal failure, infectious diseases, inflammatory bowel diseases, mental or autoimmune disorders, or terminal illnesses (life expectancy <6 months) were not included. Diagnoses of malignancy were confirmed through postoperative pathological examinations.

Baseline nutritional status was assessed using a comprehensive anthropometric, biochemical, clinical and dietary parameters evaluation. Detailed information regarding comorbidities, socio-demographic characteristics, family and personal medical history, lifestyle habits, and psychological well-being was collected, alongside current and past risk factors such as diet, smoking and physical activity.

The Karnofsky Performance Scale Index, a tool for measuring functional impairment, was determined for all study populations.

Additionally, for 60 newly diagnosed CRC patients, AT and cancer biopsies were collected during tumor resection surgery.

The study was approved by the Ethics Committee of the University of Campania “L. Vanvitelli” (No. 0016436/2020 and No. 187/2020). Before enrolment, all participants gave written informed consent.

### Anthropometric and metabolic-nutritional evaluation

At admission, two days prior to surgery, patients underwent blood sampling and an assessment of anthropometric and metabolic-nutritional parameters. Anthropometric measurements include BMI, Fat-Free Mass (FFM), waist-to-hip ratio (WHR), the bioelectrical impedance BIA, Body Cell Mass (BCM), and Body Cell Mass Index (BCMI).

For each patient, weight and height were measured by standard techniques, and BMI was calculated as weight in kilograms divided by the square of height in meters.

FFM refers to the total mass of the body excluding all fat tissue and is an important indicator of lean tissue mass, primarily muscle, which is crucial for metabolism, physical function, and overall health [81, 82].

Waist circumference was measured at the midpoint between the lower rib margin and the iliac crest (most commonly at the umbilical level), while hip circumference was measured at the level of the greater trochanter. The waist-to-hip ratio (WHR) was determined by measuring both to the nearest 0.5 cm with a plastic tape measure.

Baseline blood pressure was assessed using a standard mercury sphygmomanometer, with the disappearance of sound (phase V) used as the diastolic reading. Each measurement was taken three times, at two-minute intervals, with the patient in a supine position, on three separate occasions. The average of the last two recorded values was used for analysis. Body composition, including fat mass, fat-free mass, lean mass, muscle mass, and phase angle, was assessed using the bioelectrical impedance device BIA 101 BIVA (Akern/RJL, Florence, Italy). Measurements were performed with the participant in a relaxed supine position, ensuring that the arms and legs did not touch the body. The legs were positioned at a 45° angle, and the arms at 30° from the torso. Sensing electrodes were placed on the right wrist and ankle, while current electrodes were placed on the metacarpals and metatarsals.

Based on BIA measurements, body cell mass (BCM), an indicator of malnutrition status, was calculated. BCM represents the free fat mass directly involved in all the metabolic processes of the body, given by all the muscle tissues (about 60%), intra and extracellular water (including the water hydrating adipose tissue), internal organs (20%) and bone tissue. The Body Cell Mass Index (BCMI), a prognostic index of (mal)nutrition, inflammation and muscle mass status, used to assess muscle depletion in clinical populations [83], was also calculated using the formula: BCMI = BCM/height^2^.

The prognostic nutritional index (PNI), an indicator of the nutritional and immune status of cancer patients [84], was determined using the formula: PNI = 10 × serum albumin (g/dL) + 0.005 × total lymphocyte count (per mm^3^).

Nutritional status was evaluated using the Mini Nutritional Assessment (MNA), a validated tool that incorporates anthropometric measures (BMI, weight loss, arm and calf circumferences), general assessment (lifestyle, medication use, mobility, and the presence of depressive or cognitive symptoms), dietary assessment (number of meals, food and fluid intake, eating autonomy), and a subjective evaluation of health and nutritional status. Based on the total score, individuals were categorized into three groups: malnourished (score <17), at risk of malnutrition (score 17–23.5), and normal nutritional status (score ≥24–30) [85].

Nutritional risk was assessed using the Nutritional Risk Score, which takes into account both subjective and objective parameters, including medical history, dietary intake (covering energy and protein balance), physical examination, anthropometric measurements, quality of life, functional and mental health assessments, medication use, and laboratory parameters. Each parameter was scored from 0 to 3, with a total score of ≥3 indicating that the patient was at risk of malnutrition or already malnourished, thus requiring nutritional intervention [86].

Adherence to the Mediterranean diet was assessed using the Mediterranean Diet Adherence Screener (MEDAS), a 14-point questionnaire evaluating the frequency of consumption of specific food groups, including fruits, vegetables, legumes, olive oil, fish, and nuts [87].

The Short Physical Performance Battery (SPPB) was used to assess lower extremity function in older adults through a series of physical performance tests [88].

### Tissue biospecimen collection and processing

Visceral adipose tissue (VAT) and tumor specimens were obtained from CRC patients during surgery for tumor resection. For each CRC patient, the following specimen samples were obtained:

1) a sample of intra-tumoral-VAT (as close as possible to neoplastic lesion and within 5 cm from tumor, identified as Intra-VAT); 2) a sample of peritumoral-VAT (as close as possible to neoplastic lesion and within 15 cm from tumor, labeled as Peri-VAT); 3) VAT (at least 15 cm from neoplastic lesion, named HVAT); 4) a sample of subcutaneous adipose tissue (SAT); 5) CRC tissue (CRCT) and 6) the adjacent normal tissue (HCT).

The biopsy specimens for each patient were quickly divided into three parts, snap frozen in liquid nitrogen, and stored at −80°C.

Each biopsy specimen was disrupted and homogenized using TissueLyser LT (Qiagen) for subsequent isolation of DNA, RNA, or protein. Tissue samples were homogenized in the appropriate lysis buffers in the presence of Stainless-Steel Beads, 5 mm (cat. no. 69989, Qiagen), according to the manufacturer’s instructions.

### PBMC isolation

Blood samples were collected in ethylenediaminetetraacetic acid (EDTA) tubes.

Human peripheral blood cells (PBMC) were isolated by density gradient centrifugation over Ficoll-Hypaque (GE Healthcare, Chicago, Illinois, USA). After centrifugation at 400 g for 20 minutes, the buffy coat was carefully collected and transferred to a new Falcon tube. It was then washed twice with PBS and centrifuged at 300 g for 10 min at 4 °C.

### RNA extraction

Total RNA was extracted from PBMC samples and biopsy specimens using Trizol. After the addition of 1-bromo-3-chloropropane (Cat # B9673; Sigma), the samples were centrifuged at 12,000 rpm for 15 min at 4 °C. The aqueous upper phase was recovered and an equal volume of isopropanol was added. After 2 h of incubation at -80 °C, the samples were centrifuged at 12,000 rpm for 30 min at 4 °C and washed in ethanol. The extracted total RNA was quantified using a QIAexpert spectrophotometer (Cat #1038703; Qiagen).

### miR Quantitative Real-Time

A total of 10 ng of total RNA were reverse transcribed using TaqMan Advanced miRNA cDNA Synthesis Kit (cat. #A28007, Thermo Fisher Scientific, Massachusetts, USA) and following manufacturer instructions.

The relative quantification was performed on a Rotor-Gene Q (Qiagen), using TaqMan advanced miRNA assay (cat. #A25576, Thermo Fisher Scientific) and the TaqMan fast advanced master mix (cat. #4444963, Thermo Fisher Scientific), according to manufacturer’s instructions.

For hsa-miR21-5p, hsa-miR-92a-3p, hsa-miR-181b-5p, hsa-miR-203a-5p, hsa-miR-23a-5p, hsa-miR-1246 the following TaqMan advanced miRNA assays were used: ID: 477975_mir; ID: 477827_mir; ID: 478583_mir; ID: 478756_mir; ID: 478782_mir; 483023_mir hsa-let-7g-5p (ID: 478580_mir) was used as housekeeping microRNA. The cycling conditions were 3 min at 95 °C, followed by 40 cycles of 5 s at 95 °C and 30 s at 60 °C. The expression levels were measured in duplicate and normalized with housekeeping miR, using the 2^^-ΔCt^ method. All the experiments were performed three times.

### Real-time quantitative PCR (rt-qPCR)

1 µg of total RNA was reverse-transcribed using QuantiTect Reverse Transcription Kit (# 205310, Qiagen, Hilden, Germany). mRNA level quantification was determined on a Rotor-GENE Q (Qiagen), by using qPCR with Green-2 Go qPCR master mix (# QPCR004-5 Biobasic, Milan, Italy). The cycling conditions were 3 min at 95 °C, followed by 40 cycles of 5 s at 95 °C and 30 s at 60 °C. The relative gene expression was measured with the 2^−ΔCt^ method; the analysis was carried out through three individual experiments. Actin beta (ACTB) was used as a reference gene.

Validated Primer Assay (IDT) was used to detect expression of the following genes: IL-8 (Hs.PT.58.39926886.g), IL-10 (Hs.PT.58.2807216), IL-15 (Hs.PT.58.40896236), TGFβ (Hs.PT.58.39813975), collagen (COL1A1- Hs.PT.58.15517795), UCP-1 (Hs.PT.58.39157006), ADIPOQ (Hs.PT.58.26002735), Pref-1 (DLK1) (Hs.PT.58.40622309), PON3 (Hs.PT.584385010), TMEM26 (Hs.PT.58.4285425). For reference gene, PCR oligo pairs were used: ACTB: fw 5′-CATCCGCAAAGACCTGTACG-3′, rv 5′-CCTGCTTGC TGATCCACATC-3′.

### Protein extraction and Western blot

To 100 mg of adipose tissue biopsy, 0.5 mL of RIPA buffer (without Triton X-100) containing protease inhibitor was added. The tissue was homogenized using the Tissue-Lyser LT (Qiagen) with stainless steel beads at maximum frequency for 3-5 min until the solution became clear. The homogenate was then centrifuged at 6,000 x g for 15 min at 4 °C. After removing the fat cake (white lipid layer on top of the aqueous layer) and resuspending the loose pellet, Triton X-100 was added, and the samples were incubated at 4 °C overnight. Following centrifugation at 12,000 x g for 15 min at 4 °C, the lipid layer was discarded, and the supernatant was transferred to a new 1.5 ml tube. These steps were repeated twice to remove as much lipid as possible.

Forty μg of protein samples were separated by 10% SDS-PAGE and transferred to 0.22 μm PVDF membranes. The membranes were blocked with 5% nonfat milk in TBS-T (Tris-buffered pH8/0.15% Tween 20) at room temperature for 1 h, followed by incubation with the following primary antibodies (diluted in TBS-T): UCP-1 (Invitrogen, cat# MA5-31534), DLK-1 (Pref-1) (Elab science, cat# 18255), PON3 (Invitrogen, Cat #PA5-82512), Caspase 3 (Abcam cat#ab32351), TMEM26 (Invitrogen, cat# PA5-23477), COL1A1 (Elab science, cat# dq0217). Vinculin (Abcam, Cat #ab129002) was used for normalization.

After three washes in TBS-T, the membranes were incubated with the corresponding secondary antibodies, goat anti-rabbit IgG-h+HRP conjugated (Bethyl cat# A120-101p) and donkey anti-mouse IgG-h-I HRP conjugated (Bethyl cat# A90-137p), both diluted 1:5000, for 1 hour at room temperature. Immunocomplexes were visualized using Clarity Max Western ECL Substrate (Bio-Rad, cat#1705062). Images were captured using the Image Lab 5.2.1 software with the Molecular Imager ChemiDoc XRS Imaging System (Bio-Rad). Band densities were quantified using ImageJ 1.52n software (National Institutes of Health, Bethesda, MD, USA).

The densitometric analysis was performed using 3 separate experiments. The uncropped western blots were shown in Supplemental Material.

### Libraries preparation and sequencing

DNA was extracted from the tissues of CRC patients (three patients, six samples for each patient: Intra-VAT, Peri-VAT, HVAT, SAT, CRCT, HCT) by homogenization using the Tissue Lyser LT (Qiagen), and the tissues were resuspended in TE buffer (0.2 M Tris-HCl, 20 mM EDTA). Next, DNA was quantified via Qubit, using a buffer (198 μL) with 1 μL of dye and 1 μL of sample, after calibrating the instrument with the Life Technologies Qubit® RNA Broad-Range Assay Kit. DNA quality was assessed using TapeStation, which provides an assessment of DNA fragmentation and integrity. DNA libraries were prepared using the OncoDEEP DNA kit (CE-IVD - Charleroi, Belgium), which involves the use of the ODDX@7 panel (638 genes). Library preparation procedure included DNA fragmentation, end repair, adapter ligation, and target-specific gene amplification by PCR. The DNA libraries thus prepared were subjected to cleaning to remove any contaminants, and then a quantification followed, to ensure an adequate concentration before sequencing. Subsequently, DNA libraries were loaded onto the Novaseq 6000 Illumina platform for sequencing. The resulting sequencing data were analyzed using R software to identify genetic variants, germline and somatic mutations, and other cancer-relevant modifications. The results of the analysis were interpreted in the context of current knowledge of cancer biology and personalized therapies. The specific instructions provided with the kits used were carefully followed and standard DNA manipulation protocol were taken into account, to ensure accurate and reproducible results.

### Gene data processing

To detect variants, cDNA data were pre-processed on the ONCOKDM platform, reporting the total number of mutations and the corresponding metadata, such as location, frequency, and possible biological impact. In particular, 5263 variants were reported for the three patients and six samples per patient.

These data were the starting point for the analysis of the variants, mainly submitted to a matching with the most relevant and updated official generic or CRC variants databases or archives such as NCBI ClinVar (https://www.ncbi.nlm.nih.gov/clinvar), Human Gene Mutation (https://www.hgmd.cf.ac.uk/ac/all.php) or Leiden Open Variation Database (http://www.lovd.nl). Many papers were also examined, for an instance “Standards and Guidelines for the Interpretation and Reporting of Sequence Variants in Cancer” [89] and “ Clinical Interpretation of Sequence Variants” [90]. Benign and likely benign variants were discarded, so only 1020 variants were considered interesting and furtherly investigated.

### DNA extraction and methylation analysis

DNA was extracted from PBMC samples using the QIAamp DNA Blood Mini Kit (#51104, Qiagen) following the manufacturer’s instructions.

A total of 350 ng of the extracted DNA underwent bisulfite conversion with the EpiTect Fast DNA Bisulfite Kit (#59824, Qiagen) as per the provided protocol. Methylation analysis was carried out using pyrosequencing on the PyroMark Q48 Autoprep (Qiagen). The bisulfite-modified DNA was amplified via polymerase chain reaction (PCR) with the PyroMark PCR Kit (#978703, Qiagen), with each reaction containing 2 μL of bisulfite-treated DNA, 12.5 μL of PyroMark PCR Master Mix 2X, which includes Hot Start Tag DNA Polymerase, 2.5 μL of Coral Load Concentration 10X, and 2.5 μL of PCR primer. The amplification followed the conditions: 1 cycle at 95 °C for 15 min, followed by 40 cycles of 94°C for 30 seconds, 56 °C for 30 s, and 72 °C for 10 min. After amplification, the PCR products were separated by electrophoresis on a 2% agarose gel (Amersham Biosciences, UK). The biotinylated PCR products were then sequenced using the PyroMark Q48 Advanced CpG Reagent (#974022, Qiagen) and analyzed with the Pyro-Mark CpG SW 1.0 software (Qiagen). Methylation levels of the PON3 gene were assessed using the commercially available Hs_PON3_01_PM PyroMark CpG assay (PM00029281), targeting Island 1 in the gene promoter region (bp 95025901–95025926, CRCh37/hg19). Methylation analysis derived from 3 independent experiments.

### Statistical analysis

Results were expressed as the means ± Standard deviation. The sample size was estimated on an IBM computer by G-POWER software to investigate the difference between groups. The SAMPLE SIZE ESTIMATION (SSE) was calculated by considering its main outcome, i.e. the differences in gene expression, protein expression, genetic mutation, epigenetic markers expression in adipose tissues and tumor tissues. The sample size provides 81% power to detect a mean difference of 0.5, with an estimated standard deviation of differences of 1.0, and a significance level (alpha) of 0.005, using a two-sided paired t-test. The differences between groups were analyzed using a one-way analysis of variance (ANOVA). A p-value of < 0.05 was considered indicative of statistically significant differences between mean values. Correlations were assessed using Pearson’s R. All statistical analyses were conducted using SPSS version 26 software (Chicago, IL, USA).

## Supplementary information

Supplementary Materials

Original western blotting

## Data Availability

Any additional information and materials in this paper are available from the corresponding author upon reasonable request.
