# Supplementary material for: Adipose-tumor crosstalk in colorectal cancer: Identifying (Epi)genetic biomarkers for tumor progression and cachexia
Source: Cell Death Dis. 2025 Oct 6;16(1):675. doi: 10.1038/s41419-025-07982-6 (PMC12500986; doi:10.1038/s41419-025-07982-6)
Supplement: Supplementary file 1 — Supplementary Materials [file 41419_2025_7982_MOESM1_ESM.docx]

**Supplementary Materials**

**Adipose-Tumor Crosstalk in Colorectal Cancer: Identifying (Epi)Genetic Biomarkers for Tumor Progression and Cachexia**

Ada Pesapane^1^ *, Lucia Capasso^2^ *, Maria Rosaria Del Sorbo^2^, Lucia Scisciola^1^, Teresa Troiani^2^, Donato Mele^2^, Martina Franzese^1^, Armando Puocci^1^, Giovanni Tortorella^1^, Surina Surina^1^, Giacomo Fuschillo^1^, Francesco Caraglia^2^, Vincenzo De Falco^2^, Lucio Selvaggi^1^, Rosaria Anna Fontanella^1^, Fortunato Ciardiello^2^, Francesco Selvaggi^1^, Lucia Altucci^2,3,4^, Giuseppe Paolisso^1,5^, Michelangela Barbieri^1^ **, Angela Nebbioso^2,3^ **

^1^ Department of Advanced Medical and Surgical Sciences, University of Campania "Luigi Vanvitelli", Naples, Italy

^2^ Department of Precision Medicine, University of Campania “Luigi Vanvitelli”, Vico L. De Crecchio 7, 80138, Naples, Italy

^3^ Program of Medical Epigenetics, Vanvitelli Hospital, Naples, 80138, Italy.

^4^ Biogem, Molecular Biology and Genetics Research Institute, Ariano Irpino, 83031, Italy.

^5^ UniCamillus, International Medical University, Rome, Italy.

*These authors contributed equally to this work.

** These authors contributed equally to this work.

Corresponding author:

Giuseppe Paolisso

Department of Advanced Medical and Surgical Sciences, University of Campania "Luigi Vanvitelli", Naples, Italy; tel: +39 0815665134

[giuseppe.paolisso@unicampania.it](mailto:giuseppe.paolisso@unicampania.it)

| **GENE** | **FORWARD** | **REVERSE** |
| --- | --- | --- |
| ACTB | 5-CATCCGCAAAGACCTGTACG-3 | 5-CCTGCTTGCTGATCCACATC-3 |

**Supplementary Table 1 A.** Primer used as reference gene

|  | BEFORE MUTATION POINT | | AFTER MUTATION POINT | |
| --- | --- | --- | --- | --- |
| **GENE** | **FORWARD** | **REVERSE** | **FORWARD** | **REVERSE** |
| APC | 5-CAGCTCCATCCAAGTTCTGC-3 | 5-TGCTTCCTGTGTCGTCTGAT-3 | 5-ATGGCGAAAACTCCCACCTA-3 | 5-ATGTTTGCTGTGCTCACGTT-3 |
| AXIN | 5-AGAGGGAGAAATGCGTGGAT-3 | 5-CGCCTGGTCAAACATGATGG-3 | 5-ACGATACTGGACGATCACCT-3 | 5-GGATGTAGTGGTGGTGGACA-3 |
| CYLD | 5-CTGAAGAAGGCGCTGTTTGT-3 | 5-TGACCCTGGATGCCTTTCTT-3 | 5-TTCTGACCACCATCCCGATC-3 | 5-TTCTGACCACCATCCCGATC-3 |
| KRAS | 5-ACACAAAACAGGCTCAGGAC-3 | 5-TCACACAGCCAGGAGTCTTT-3 | 5-TTGTGGTAGTTGGAGCTGGT-3 | 5-CTCCTCTTGACCTGCTGTGT-3 |

**Supplementary Table 1 B.** List of primers used for RT-qPCR

| **Variables** | **miR92a** | | **miR21** | |
| --- | --- | --- | --- | --- |
|  | **r** | **p** | **r** | **p** |
| Body fat percentage | 0.247 | 0.043 | -0.156 | 0.216 |
| FFM | -0.288 | 0.013 | -0.157 | 0.190 |
| Skeletal muscle mass | -0.262 | 0.031 | -0.078 | 0.535 |
| BCM (Body Cell Mass) | -0.346 | 0.005 | -0.217 | 0.083 |
| BCMI (Body Cell Mass Index) | -0.256 | 0.048 | -0.222 | 0.097 |

**Supplementary Table 2A.** Correlation between anthropometric-metabolic parameters and miRNAs (miR92a and miR21) expression levels in PBMC in the entire study population

| **Variables** | **PON3 DNA methylation** | |
| --- | --- | --- |
|  | **r** | **p** |
| FFM | 0.318 | 0.009 |
| BCM | 0.245 | 0.048 |
| Body fat percentage | -0.320 | 0.008 |
| WHR | -0.371 | 0.007 |
| Karnofski index | 0.453 | 0.022 |
| MNA score | 0.221* | 0.058* |
| miR-92a | -0.172* | 0.079* |

**Supplementary Table 2B.** Correlation between anthropometric-metabolic parameters and PON3 DNA methylation levels in PBMC isolated from the study population

*****The correlation is significant with the methylation of position 3 of PON3 gene promoter


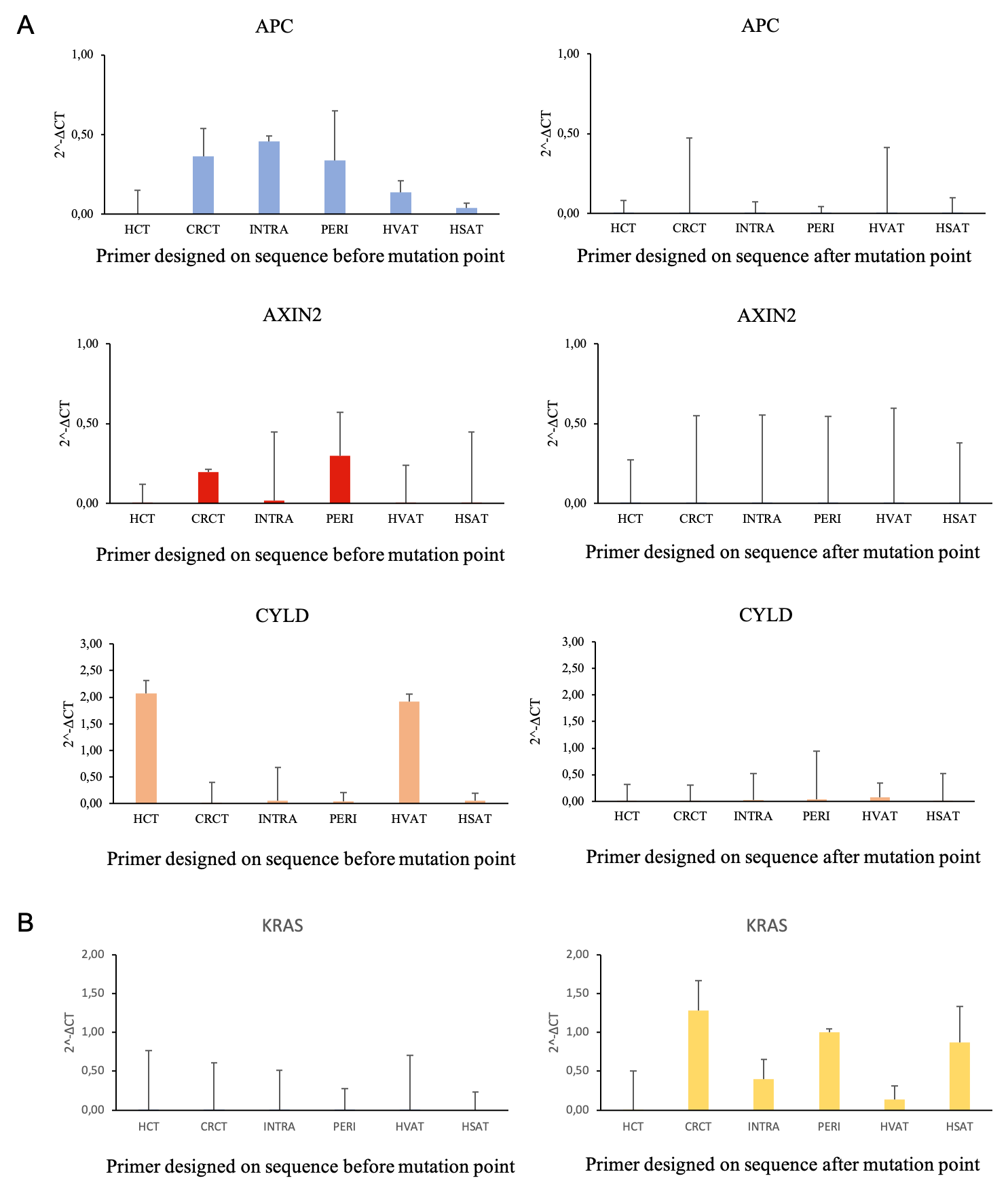

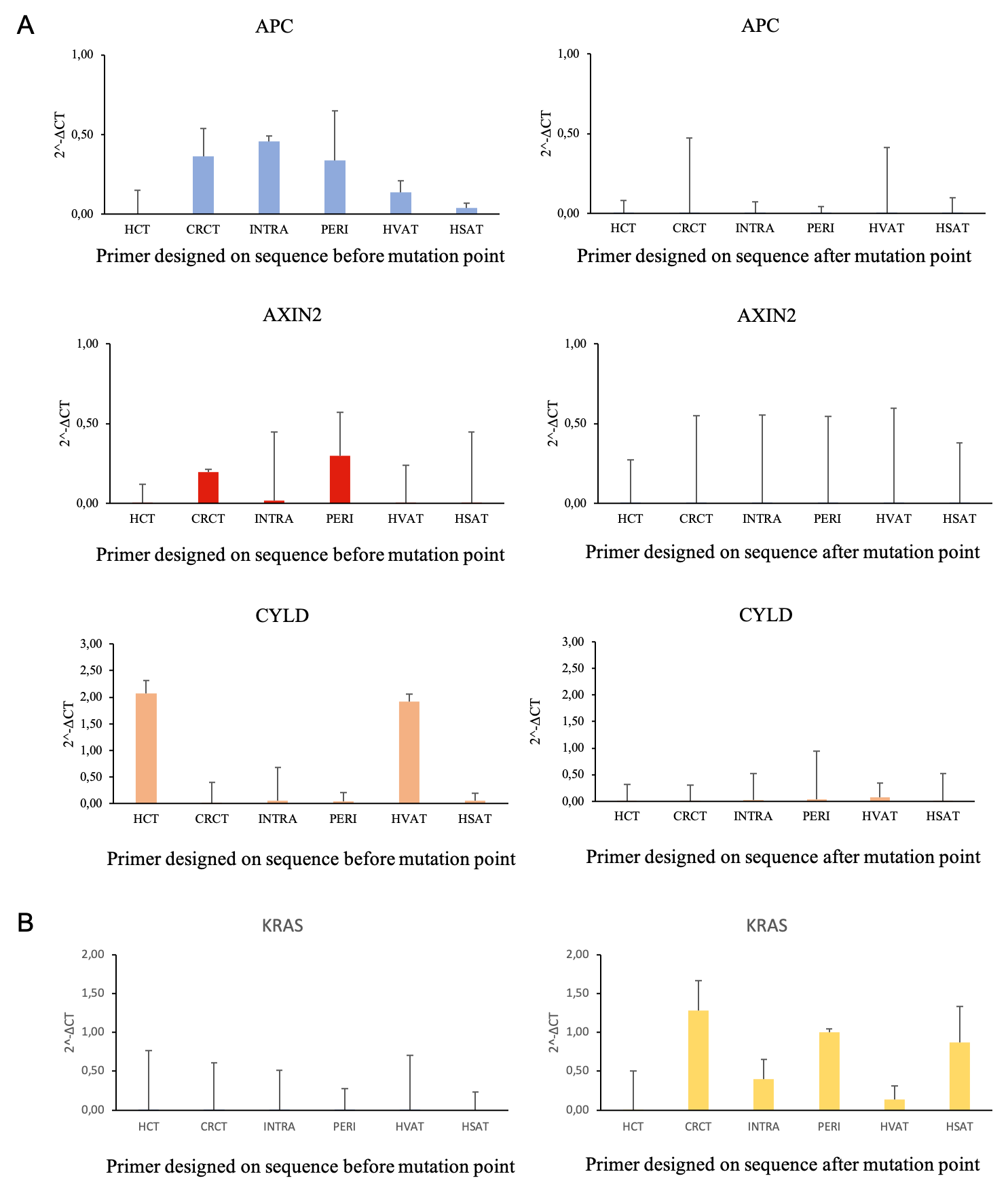


**Supplementary Figure 1**. q-PCR for APC **(A)** and KRAS **(B)** in CRCT samples using primers designed on the sequence before and after mutation point. Error bars indicate standard deviation (SD) of two replicates.
