# Supplementary material for: Adipose-tumor crosstalk in colorectal cancer: Identifying (Epi)genetic biomarkers for tumor progression and cachexia
Source: Cell Death Dis. 2025 Oct 6;16(1):675. doi: 10.1038/s41419-025-07982-6 (PMC12500986; doi:10.1038/s41419-025-07982-6)
Supplement: Supplementary file 2 — Original western blotting [file 41419_2025_7982_MOESM2_ESM.pptx]

## Slide 1
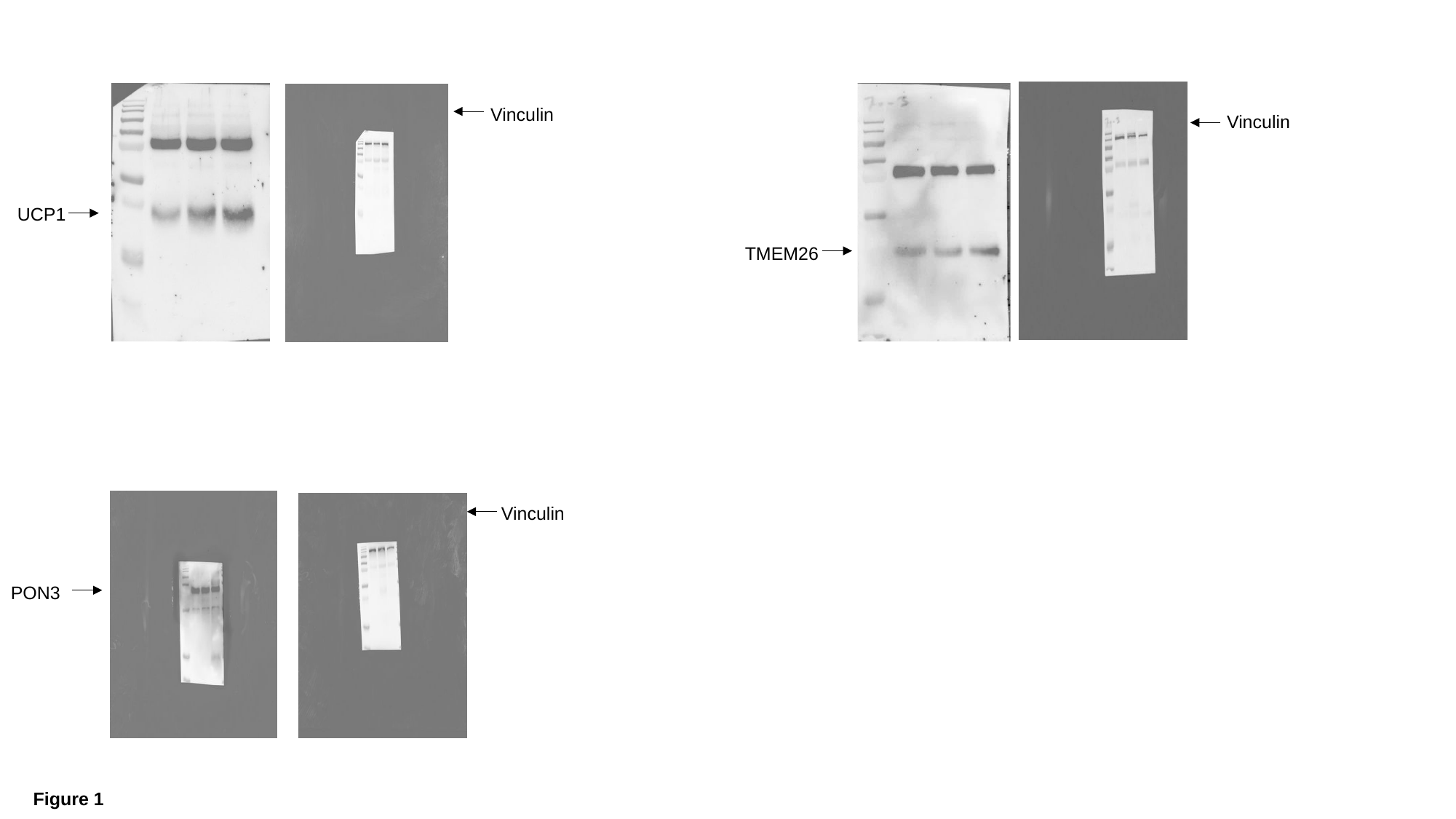

Vinculin
Vinculin
UCP1
TMEM26
Vinculin
PON3
Figure 1

## Slide 2
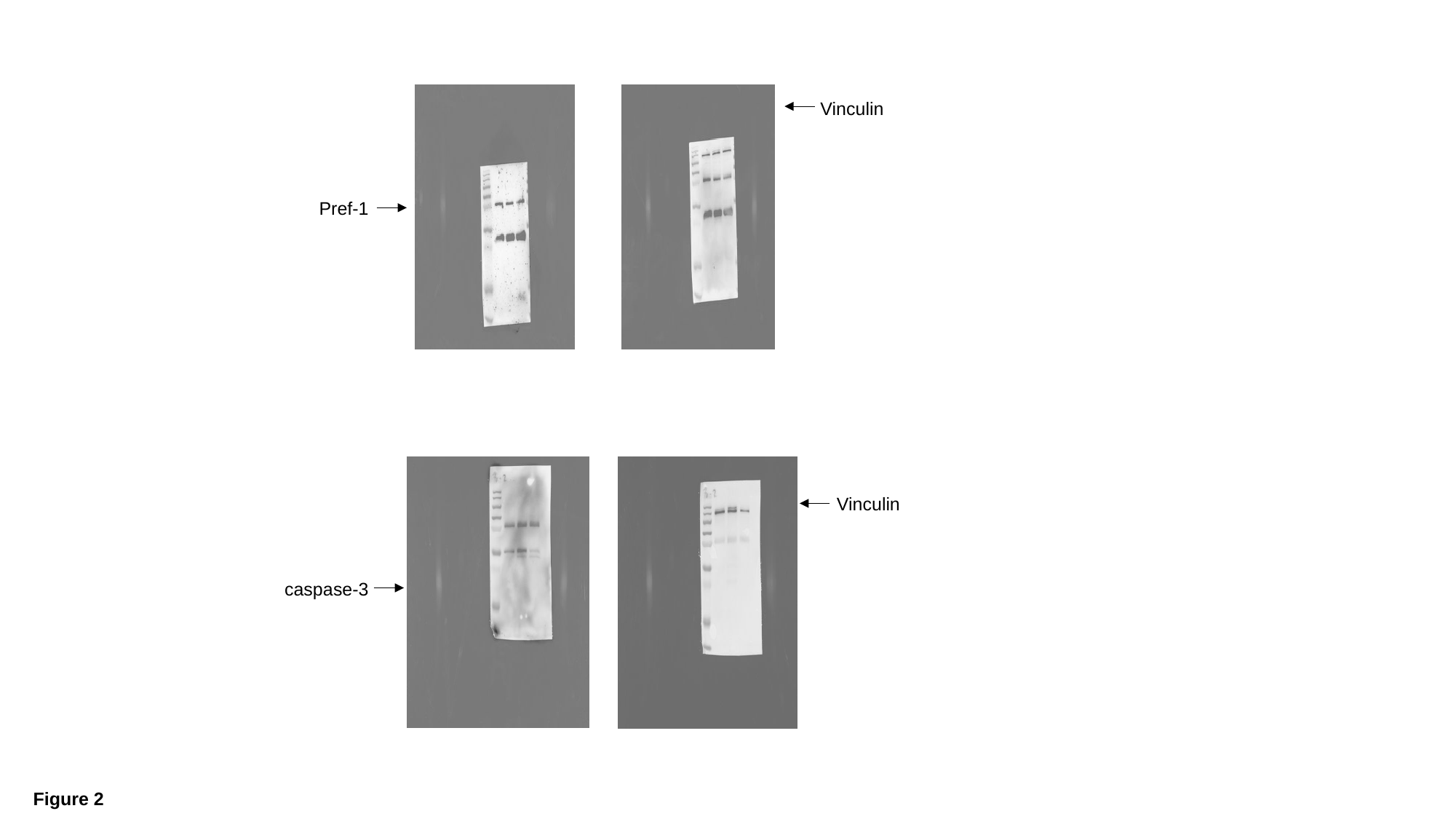

Vinculin
Pref-1
Vinculin
caspase-3
Figure 2

## Slide 3
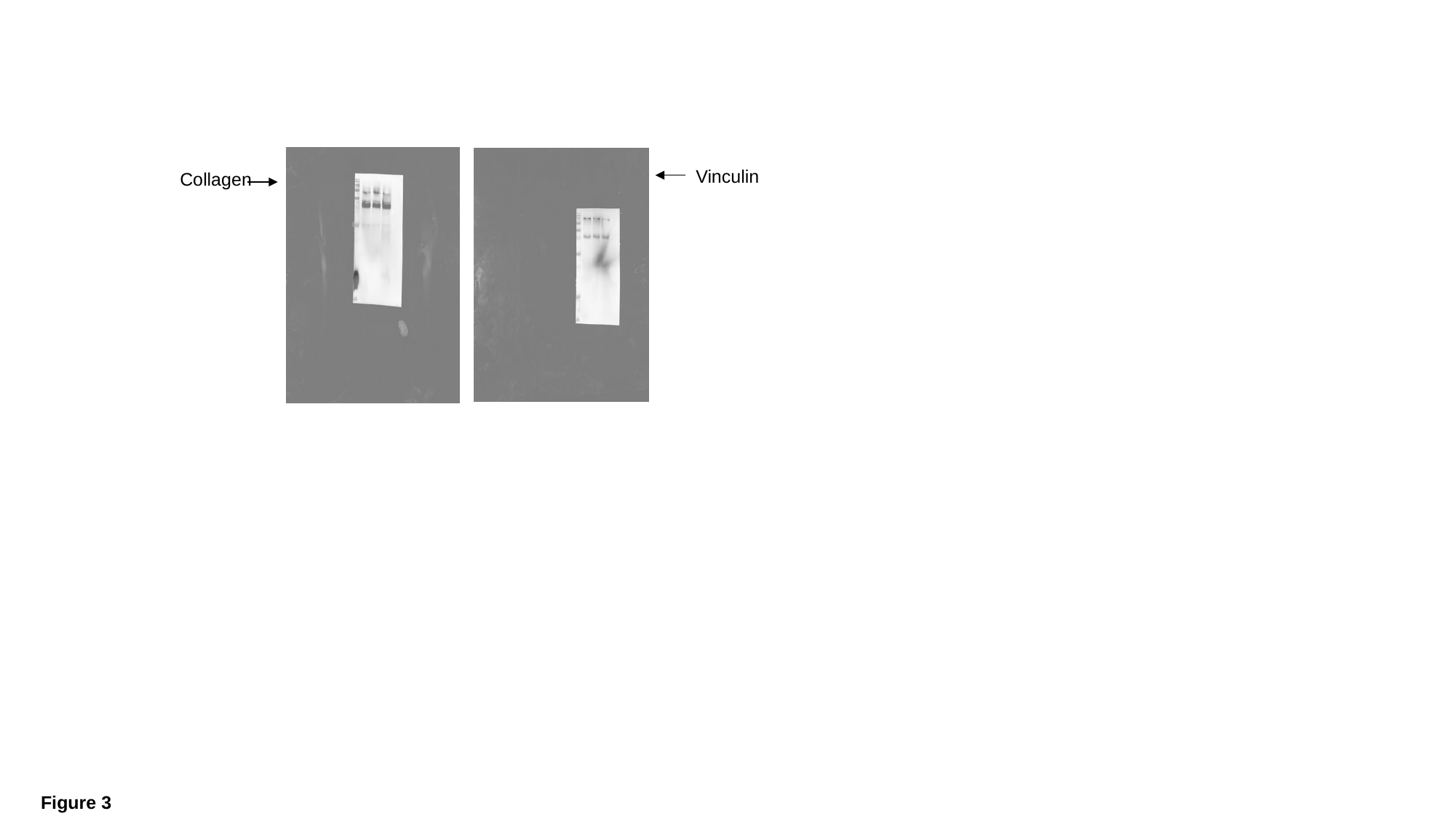

Vinculin
Collagen
Figure 3
